# *Campylobacter* Phage Isolation and Characterization: What We Have Learned So Far

**DOI:** 10.3390/mps2010018

**Published:** 2019-02-15

**Authors:** Claudia Jäckel, Jens Andre Hammerl, Stefan Hertwig

**Affiliations:** Department of Biological Safety, German Federal Institute for Risk Assessment, Max-Dohrn Str. 8-10, 10589 Berlin, Germany; claudia.jaeckel@bfr.bund.de (C.J.); jens-andre.hammerl@bfr.bund.de (J.A.H.)

**Keywords:** *Campylobacter*, bacteriophage (phage), isolation, genome, propagation, sequencing, host range

## Abstract

Lytic *Campylobacter* phages, which can be used to combat this pathogen in animals and on food products, have been studied for more than 30 years. Though, due to some peculiarities of the phages, which hampered their isolation and particularly their molecular analysis for a long time, progress in this research field was rather slow. Meanwhile, the situation has changed and much more is known about the biology and genetics of those phages. In this article, we address specific issues that should be considered when *Campylobacter* phages are studied, starting with the isolation and propagation of the phages and ending with a thorough characterization including whole-genome sequencing. The basis for advice and recommendations given here is a careful review of the scientific literature and experiences that we have had ourselves with *Campylobacter* phages.

## 1. Introduction

Campylobacteriosis is one of the most frequently reported foodborne illnesses in humans [1]. The disease is primarily caused by the thermophilic species *Campylobacter jejuni* and *Campylobacter coli* [2]. Typical symptoms of campylobacteriosis are diarrhea, cramping, abdominal pain, and fever [3]. Human infections are caused mainly by the handling, preparation, and consumption of undercooked meats, especially poultry [4]. *Campylobacter* is a commensal of the gastrointestinal tract of various mammals and birds and is frequently found in chicken flocks [5,6]. The prevalence of *Campylobacter*-positive chicken is generally high and transmission of the bacteria from bird to bird occurs rapidly [7]. Fifty to eighty percent of human infections are thought to be associated with chicken farming. Thus, reduction of *Campylobacter* in chicken could decrease the number of human campylobacteriosis significantly [8,9].

To reduce the number of *Campylobacter* along the food chain, various biosecurity measures and post-slaughter decontamination procedures have been investigated, e.g., the minimization of environmental exposure (fly nets, change of footwear), the addition of organic acids to drinking water, and the chemical or physical decontamination of carcasses [10]. However, some of these measures are expensive and not always efficient [11]. Since there is not yet any vaccine available on the market to reduce the intestinal colonization of broilers by *Campylobacter* [12], phages that have in the past been used for typing might be an appropriate means to combat this pathogen in chicken and chicken products [9,11,13,14,15,16,17,18,19,20,21]. Phage administration in the laboratory reduced *C. jejuni* colonization of the broiler gut and the contamination on chicken skin by several orders of magnitude [14,15,22,23,24,25,26,27]. Moreover, a phage cocktail applied via drinking water also efficiently reduced *Campylobacter* counts in chicken on a commercial broiler farm [28]. However, since *Campylobacter* does not grow below 31 °C, a reduction of the pathogen at low temperatures can only be achieved by very high numbers of phages causing lysis from without [16,25].

Almost all *Campylobacter* phages described so far are lytic and belong to the family Myoviridae [29]. There are some older reports on siphoviruses infecting *Campylobacter*, but little information on these phages is available [30,31]. According to their genome size and morphology, lytic *Campylobacter* phages have been divided into three groups [21]. Group I contains phages with large genomes (~320–425 kb) that seem to be rare [14,21]. They have significantly larger head dimensions than phages of the other groups [21] but have not yet been described in detail or used for applications. Moreover, to date no group I *Campylobacter* phage has been sequenced. However, due to their differences from group II and III phages, methods developed for the isolation of these groups may not be suited for group I. Thus, group I phages may be more common than expected. By contrast, members of group II (~175–183 kb) and group III (~131–135 kb) have been frequently isolated in many countries [21,29]. Common to group II and group III phages are a low burst size [15,23,32], a very low GC content of 26 to 27%, and an insensitivity to cleavage by many restriction endonucleases [33,34,35]. These properties hampered the quick identification of group II and group III phages and the molecular characterization of these phages for many years [21,22,36,37]. Up to now, three group II (CP21, CP220, and CPt10) [37,38] and eight group III (CP81, CP30A, NCTC12673, PC14, PC5, vB_CjeM_Los1, CPX (CPX (NC_016562): [39]), and CP8) phages have been completely sequenced [34,40,41,42,43], whereas the genome of group II phage vB_CcoM-IBB_35 could only partially been determined (Table 1) [44]. Phages within each group revealed strong DNA homologies. Meanwhile, only weak similarities were found to exist between group II and group III phages, for which two new genera, “Cp220likevirus” (later renamed to CP220virus [45]) and “Cp8unalikevirus” (later renamed to CP8virus [45]), respectively, have been proposed. Both groups are distantly related to T4-like phages. While group III phages possess colinear genomes, those of group II phages are composed of large modules separated by long DNA repeat regions, which could lead to rearrangements [29,33]. Thus far, two subgroups of group II phages exhibiting a different modular genome organization and host range have been identified by PCR and sequencing [38,46]. A striking feature of group II phages is that they frequently infect both *C. jejuni* and *C. coli* [21]. However, phages of group III often lyse more *C. jejuni* strains than group II phages and may exhibit a stronger lytic activity [15,37,46]. The successive application of a group III and a group II phage reduced the numbers of *C. jejuni* in chickens most efficiently [15]. Therefore, a broad selection of well-characterized phages should be available for the reduction of *Campylobacter* in animals and food products.

Due to the fact that working with *Campylobacter* phages is a challenging task which may cause problems, we want to address a number of critical steps and give some advice how problems could be avoided or solved. Some aspects important for *Campylobacter* phage research and application have already been discussed by others [47,48,49]. These papers provide some general information and protocols on the isolation, propagation, and characterization (i.e., host range determination, protein profiling, receptor type identification, Pulsed-Field Gel Electrophoresis) of *Campylobacter* phages as well as on the use of the phages in live birds and food. Due to new insights into this subject and experiences in our laboratory, this article gives additional information on what should be noted and reviews some issues, which have not yet been considered (e.g., propagation of the phages in liquid cultures, strategies for the isolation and sequencing of the phage DNA).

## 2. Isolation of *Campylobacter* Phages

Sampling: *Campylobacter* phages are normally found in all places at which their hosts occur [50]. They have been isolated from poultry (i.e., meat, liver, skin, feces, and intestines), manure, and sewage [22,36,43,50,51,52]. High rates of *Campylobacter* phages have been isolated from organic farms [53]. Samples intended to be used for phage isolation should not be frozen because this treatment may reduce the plaque-forming ability significantly (by up to 80%) [22,54]. While *Campylobacter* phages were isolated at a rate of 11% (34/300) from the skin of fresh chicken thighs, isolation from frozen chicken thighs was unsuccessful [22,55]. Even though polymerase chain reaction (PCR) analysis revealed the presence of *Campylobacter* phages in many frozen food samples, most of the detected phages did not show any lytic activity [54]. Some *Campylobacter* phages seem to be unstable and could not be successfully propagated after isolation [56]. Therefore, rough treatments (e.g., extensive vortexing) should be avoided.

Sample preparation: Solid sample material can be incubated in Sodium chloride/Magnesium sulfate (SM)-buffer to resuspend phages. On chicken skin, *Campylobacter* phages are able to adhere very tightly. They can be removed by rinsing and swabbing but the use of a Stomacher^®^ (i.e. BagMixer^®^, Interscience, Saint Nom, France) may give the best results. After centrifugation (10,000 × *g*, 20–60 min) of the material and subsequent filtration (0.45 µm and 0.22 µm) of the supernatant, samples can be examined for lytic activity. A 10-fold concentration of phage preparations prior to spotting on indicator strains can be achieved by centrifugal filter units (i.e., Amicon^®^ Ultra Centrifugal Filters, Merck KGaA, Darmstadt, Germany, Vivaspin^®^, Sartorius, Göttingen, Germany).

(Pre-)screening for *Campylobacter* phages by PCR: PCR may be useful to pre-screen samples and to detect and discriminate group II and group III phages quickly. We applied the method to detect *Campylobacter* phages in various meat products and samples collected from chicken and pig farms. In 50 out of 110 samples (45.5%), group II and/or group III phages were identified. PCR-positive samples which do not show lytic activity on common indicator strains can then be examined with other strains that may be suitable hosts for the phage [54]. On the other hand, PCR-negative samples may contain unusual (e.g., group I) *Campylobacter* phages and should also be examined for lytic activity.

Selection of indicator strains: *Campylobacter jejuni* NCTC12662 (PT14) is often used for testing because it is susceptible to a wide range of phages [35,50,57,58]. Nevertheless, it is recommendable to study a broad range of strains representing several *fla*-types and Penner serotypes since group II and group III phages bind to different cell receptors, the flagellum and capsular polysaccharide, respectively [21,34,48,57,58,59,60]. It is conceivable that the fact that such similar *Campylobacter* phages have been isolated it is, *inter alia*, caused by the selection of the same indicator strains. A protocol is available to determine the receptor dependency of *Campylobacter* phages [48].

Cultivation of indicator strains: Cultures of indicator strains should be created in flasks rather than tubes, as growth of *Campylobacter* is enhanced by a large surface for gas exchange. While different media (e.g., Mueller–Hinton, Brain Heart Infusion, *Brucella* broth) can be used to cultivate *Campylobacter* [61], the right choice of overlay (soft agar) is important for the outcome of activity tests. The agar should contain CaCl_2_ and MgSO_4_, which are thought to facilitate the attachment of phages to their host cell, even though in some cases the addition of divalent cations may be counter-productive. As did other groups, we obtained a dense and even lawn of *Campylobacter* on NZCYM agar [21,62,63]. By contrast, e.g., lysogeny broth (LB) is less suited for this application because of an uneven and slimy growth of bacteria, which impedes the detection of plaques.

Determination of lytic activity: The lytic activity of phages can not only be determined by plaque assays but also by a microplate test that allows the rapid, cheaper, and less time-consuming identification of susceptible *Campylobacter* strains [64]. However, cross-contamination of strains and/or phages may be enhanced by this method. Upon the detection of lytic activity, dilutions of phage preparations can be plated to obtain single plaques. Plaques produced by lytic *Campylobacter* phages are typically small (~1 mm in diameter) and slightly turbid. Since inhibition zones in the bacterial lawn can also be caused by other substances, e.g., bacteriocins [53], a zoom stereo microscope may be helpful to detect and count plaques. Individual phages should then be recovered by three consecutive single plaque isolations [34,46].

## 3. Propagation, Concentration, and Purification of the Phages

Propagation: The propagation of lytic phages is generally performed by the production of agar plates exhibiting confluent lysis or by the infection of bacterial cultures [63]. Both methods can be used for *Campylobacter* phages but they are not similarly suited for all phages [34,46,47,48]. Thus, for each phage the optimal procedure should be determined. Phage propagation in liquid cultures is somewhat more demanding because the growth phase of the bacteria at which phages are added and the MOI (multiplicity of infection) have to be determined to obtain maximal phage titers [63]. In our laboratory we mostly achieved the best results (˃10^8^ pfu/mL) by infecting 100-mL cultures of the indicator strain (OD_588_ of ~0.4) with phages at an MOI of ~0.01 followed by incubation for 12–24 h at 42 °C [15,34,38,46]. Very long incubation periods may give lower titers due to the binding of phages to cell debris [65]. Although *Campylobacter* is a microaerophile, it grows well under soft shaking (100 rpm) in a cell culture flask with a filter cap placed in a box containing a gas-generating sachet to approximate optimal growth conditions (10% CO_2_, 5% O_2_, 85% N_2_). This approach has the advantage that the mass lysate does not contain any contaminating agar, which may aggravate filtration. However, a procedure has been described in which soft-agar is not harvested but overlaid with buffer that is removed from the plates after incubation overnight [36,47,50].

Concentration: After the removal of the remaining cells, debris, and, if necessary, agar by centrifugation (10,000 × *g*, 20 min), the lysate is filtrated (0.45 and 0.22 µm). To degrade bacterial DNA and RNA, 20 mg/mL DNase and RNase should be added to the lysate, which should be incubated for 30 to 60 min at 37 °C. Thereafter, phage particles can be concentrated by ammonium acetate purification [66], ultracentrifugation, polyethylene glycol (PEG) precipitation, tangential filtration, or the use of centrifugal concentrators (i.e., Vivaspin^®,^ Sartorius, Göttingen, Germany, Nanosep Centrifugal Devices^®^, Pall Corporation, New York, USA) depending on the volume of the lysate and the available technological equipment [34,36,46,50]. We mostly prefer concentration by an ultracentrifuge using a rotor for six tubes that each can be filled with approximately 100 mL lysate. After centrifugation for 2 hours (100,000 to 200,000 × *g*) at 10 °C, almost all phage particles are sedimented and the pellet can be resuspended in 1–2 mL of SM-buffer. PEG precipitation is a cheap and simple method to harvest phages, which is suited for large volumes [63]. However, pellets obtained by the centrifugation of precipitated particles are rather dirty and may cause problems during the purification of phages by density gradient centrifugation. Commercial centrifugal concentrators are also simple in use but are mainly suited for small volumes (less than 100 mL) as the membrane is rather quickly blocked by phage particles and other ingredients of the lysate (e.g., agar remnants), which may pollute the retentate.

Purification and electron microscopic analysis: For the purification of *Campylobacter* phages by CsCl_2_ density gradient centrifugation, standard protocols can be used. To obtain a clearly visible phage band, at least 10^9^ infectious particles should be applied. After pulling out the phage band with a syringe, cesium chloride can be removed by dialysis or by the use of centrifugal filter units. Thereafter, the preparation can be utilized for various studies, e.g., the determination of the morphology of the phages by electron microscopy, the host range, or the analysis of the phage genome and structural proteins. Electron micrographs are usually taken from virions negatively stained with uranyl acetate. However, to determine the exact size of particles and to visualize detailed structures of the phages, staining with phosphotungstate, sodium silicotungstate, or ammonium molybdate may be advisable [29,34,35]. *Campylobacter* phages can be classified according their morphology, but since group II and group III phages have similar outlines and dimensions, discrimination necessitates high-quality electron micrographs [29].

## 4. Isolation and Analysis of *Campylobacter* Phage DNA

DNA extraction: All *Campylobacter* phages that have yet been sequenced possess double-stranded DNA [29,33]. However, there is a striking difference between the DNA of group II and group III phages, which should be taken into account when phage DNA is prepared [34,46]. While the standard protocol [63], which includes phenol-chloroform extractions, is suitable for the isolation of group II phage DNA, it fails with the DNA of group III phages because of the unusual behavior of the DNA—it remains associated with the interphase, possibly caused by tightly bound protein [29,34,46]. Therefore, phenol-chloroform extractions should be omitted when the DNA of group III phages is isolated. In this case, it is recommendable to digest the phage preparation with proteinase K and sodium dodecyl sulfate (SDS) followed by the precipitation of the DNA with alcohol [34]. Alternatively, commercially available phage DNA isolation kits can be used. The quantification of *Campylobacter* phage DNA by spectrophotometric methods may give inaccurate results [41].

Restriction analysis and PFGE: *Campylobacter* phage DNA is highly resistant against digestion by many restriction endonucleases (i.e., AvaII, BamHI, ClaI, EcoRI, EcoRV, HaeIII, HindIII, HinfI, HpaII, PstI, PvuI RsaI, ScaI) [21]. To roughly determine the genome size of the phages and to allocate them to groups and subgroups, PFGE analyses were performed using the restriction enzyme HhaI, for which a detailed protocol has been published [48]. HhaI recognizes the site 5′-GCGC-3′ but cleaves *Campylobacter* phage DNA only rarely [35]. The DNA of *Campylobacter* phages is hardly cleaved by enzymes whose recognition sites contain the bases cytosine and guanine because of a yet unknown modification of the DNA. By contrast, restriction endonucleases, which recognize sheer A/T sequences (e.g., DraI, SmiI, VspI) can be used to cut the phage DNA and to compare restriction patterns on standard agarose gels allowing a cost-efficient and time-saving analysis [34,36].

Phage DNA amplification: It has been reported that *Campylobacter* phage DNA was refractory to PCR amplification [37]. However, using the DreamTaq DNA polymerase amplification components (Thermo Fisher Scientific, Waltham, Massachusetts, USA), we never faced this problem, neither with group II nor with group III phages. To amplify the long repeat regions of group II phages for sequencing, primers up to 40 nucleotides in length were used. The genomes of *Campylobacter* phages can also be amplified with a whole-genome amplification kit yielding high amounts of DNA that can be used for further studies (e.g., restriction analyses, DNA–DNA hybridization, PCR) [34].

Genome sequencing: The sequencing of *Campylobacter* phage genomes is a challenging task. Besides modifications of the DNA, which can impede PCR reactions, the low G + C content (~27%) and extensive repetitive sequences complicate whole-genome sequencing. Thus, due to substances that inhibited Taq and ϕ29 polymerases, only five contigs of the genome of group II phage vB_CcoM-IBB_35 could be obtained [44]. To date, all *Campylobacter* phage genomes have been sequenced by short-read sequencing, which is prone to homopolymer errors. In addition, long DNA repeats, as they occur in group II phages, obstruct the assembly of reads. To sequence the repeats of phage CP21, the respective regions were amplified by PCR and used as targets for in vitro transposon mutagenesis. Upon the molecular cloning of the marked PCR products in *Escherichia coli*, transformants with transposon insertions at different positions within each repeat were sequenced to determine the whole sequences of the regions [46]. Long-read sequencing (i.e., PacBio, MinION) can solve some problems but often requires high amounts of DNA (2–10 µg), which cannot be easily prepared from *Campylobacter* phages. The use of a whole-genome amplification kit may provide enough DNA but the amplified DNA usually contains some nucleotide exchanges and is therefore not equivalent to native DNA [34].

## 5. Studies Important for the Application of *Campylobacter* Phages

Phages intended to be used for the control of pathogens have to fulfill a number of requirements [34]. Phage genomes, for example, have to be free from undesired genes, e.g., genes encoding toxins. Although many gene products of the hitherto sequenced *Campylobacter* phages could not be functionally assigned, virulence-associated genes have not been detected on their genomes [34,40,41,42,43]. The genomes are linear, circularly permuted molecules. It cannot be excluded that the phages are able to transduce DNA, but this has yet to be examined. To harness phages they should be stable against a wide range of pH values and temperatures [26]. A broad host range is another important prerequisite for successful application. As mentioned above, group II and group III phages diverge in terms of their host specificity [21,29,33]. In addition, the kinetics of infection may be different. For that reason, phage cocktails should contain members of both groups to aim at *C. jejuni* and *C. coli*, to optimize the application strategy and to prevent phage resistance. Moreover, phages within each group may differ in their host range and lytic activity, even though they are genetically very similar. Group II phages, e.g., could be allocated to two subgroups, one of which lysed nearly twice as many strains than the other [46]. A comparison of group III phages revealed that one phage reduced the number of *Campylobacter* in vitro significantly more strongly than the remaining phages and was therefore chosen for an animal experiment [15]. This and other studies also showed that individual *Campylobacter* phages may induce different rates of resistance. The reason for this phenomenon is not only the fact that group II and group III phages use different host cell receptors. Even within each group, resistance rates may vary. Therefore, a number of *Campylobacter* phages and a wide spectrum of strains covering various Penner serotypes and *fla*-types should be examined to find the best phage candidates for applications. It must, however, be taken into account that data that have been collected in vitro cannot simply be transferred to real conditions (e.g., the gut of a chicken) where physicochemical factors and the resident microflora may influence the outcome of the application.

## 6. Conclusions

**Figure 1:**
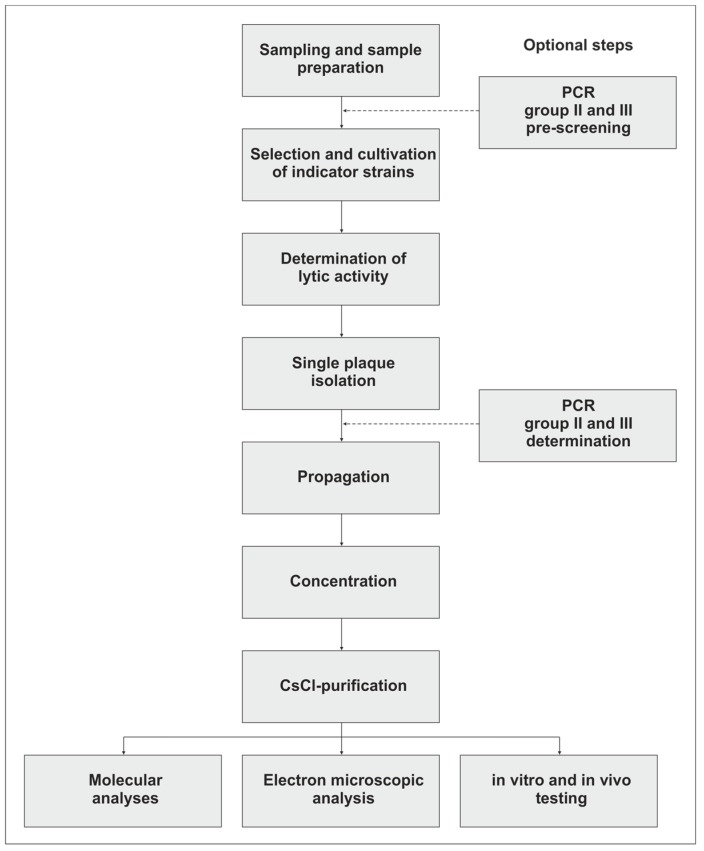
Flowchart of *Campylobacter* phage isolation, propagation, purification, and analysis. PCR: Polymerase chain reaction.

*Campylobacter* is an important foodborne pathogen which can be reduced along the food chain by the application of lytic phages. To create efficient and safe *Campylobacter* phage cocktails, methods are needed to isolate, propagate, and purify new phages for further analyses. Compared with most other lytic phages, *Campylobacter* phages exhibit some characteristics which make their application rather difficult. However, many problems that we have faced in the past could be solved by intensive studies on these phages. Thus, protocols are now available which allow the quick detection, isolation, and characterization of *Campylobacter* phages [47,48]. The main steps of this procedure are outlined in Figure 1. Nevertheless, the question arises as to why, up to now, very similar phages have been described and whether other *Campylobacter* phages exist in nature, which may possess novel properties and could be harnessed for applications. This question can now be answered more easily since a lot more information is available on the biology and genetics of these interesting phages.

## Figures and Tables

**Table 1:** Some characteristics of hitherto sequenced *Campylobacter* phages.

Phage Group	CP220virus (Group II)				CP8virus (Group III)							
Phage	CP220	CPt10	CP21	IBB_35	CP81	NCTC 12673	CPX	CP8	CP30A	PC5	PC14	vB_CjeM_Los1
Source	Chicken	Environ- ment	Water, organic farm	Poultry ceca	Chicken skin	Poultry excreta	Retail chicken	Chicken ceca	Poultry excreta	Chicken ceca	Chicken ceca	Poultry excreta
Year of isolation	2003	1989	2011	n.a.	2008/2009	Before 1985	n.a.	n.a.	n.a.	2011/2012	2011/2012	n.a.
Country	United Kingdom	United Kingdom	Germany	Portugal	Germany	USA	United Kingdom	United Kingdom	United Kingdom	Slovenia	Slovenia	Ireland
Family	Myo- viridae	Myo- viridae	Myo- viridae	Myo- viridae	Myo- viridae	Myo- viridae	Myo- viridae	Myo- viridae	Myo- viridae	Myo-viridae	Myo- viridae	Myo- viridae
Host range	*C. jejuni*, *C. coli*	*C. jejuni*, *C. coli*	*C. jejuni*, *C. coli*	*C. jejuni*, *C. coli*	*C. jejuni*	*C. jejuni*	*C. jejuni*	*C. jejuni*	*C. jejuni*	*C. jejuni*	*C. jejuni*	*C. jejuni*
Restriction	Refractory	Refractory	Refractory	Refractory	Refractory	Refractory	Refractory	Refractory	Refractory	Refractory	Refractory	n.a.
Sequencing/ platform	Shotgun seq. DNA libraries	454 FLX pyroseq. and PCR/ Sanger	454 FLX pyroseq., and PCR/ Sanger	454 FLX pyroseq.	454 FLX pyroseq.	Fidelity System	454 FLX pyroseq.	n.a.	454 FLX pyroseq.	454 FLX pyroseq.	454 FLX pyroseq.	Illumina
Genome size (bp)	177,493	175,720	182,833	172,065	132,454	135,041	132,662	132,667	133,572	131,095	134,927	134,073
Complex repeat regions	+	+	+	n.a.	-	-	-	-	-	-	-	-
GC content (%)	27.4	27.3	27.2	27.4	26.1	26.2	26.0	26.0	26.1	26.1	26.2	26.2
PFGE size (kb)	~197	n.a.	~209	~204	~145	~170	n.a.	~140	n. a.	~150	~150	n.a.
Accession no.	FN667788	FN667789	NC019507	HM246720-4	FR823450	NC015464	NC016562.1	KF148616	NC018861	KX229736	KX236333	KX879627
Reference	[37]	[37]	[38,46]	[44]	[34]	[41]	n.a.	[43]	[40]	[42]	[42]	[43]

Abbreviations: n.a., not available; PFGE, Pulsed-Field Gel Electrophoresis.
